# Supplementary material for: Downsizing the molecular spring of the giant protein titin reveals that skeletal muscle titin determines passive stiffness and drives longitudinal hypertrophy
Source: eLife. 2018 Dec 19;7:e40532. doi: 10.7554/eLife.40532 (PMC6300359; doi:10.7554/eLife.40532)
Supplement: Transparent reporting form [file elife-40532-transrepform.docx]

***eLife’s* transparent reporting form**

We encourage authors to provide detailed information *within their submission* to facilitate the interpretation and replication of experiments. Authors can upload supporting documentation to indicate the use of appropriate reporting guidelines for health-related research (see [EQUATOR Network](http://www.equator-network.org/%20)), life science research (see the [BioSharing Information Resource](https://biosharing.org/)), or the [ARRIVE guidelines](http://www.plosbiology.org/article/info:doi/10.1371/journal.pbio.1000412) for reporting work involving animal research. Where applicable, authors should refer to any relevant reporting standards documents in this form.

If you have any questions, please consult our Journal Policies and/or contact us: [editorial@elifesciences.org](mailto:editorial@elifesciences.org).

**Sample-size estimation**

- You should state whether an appropriate sample size was computed when the study was being designed
- You should state the statistical method of sample size computation and any required assumptions
- If no explicit power analysis was used, you should describe how you decided what sample (replicate) size (number) to use

Please outline where this information can be found within the submission (e.g., sections or figure legends), or explain why this information doesn’t apply to your submission:

## The number of animals needed for the various experiments was calculated wherever possible based on power calculations, with the significance level set at *p*<0.01, and assuming variance similar to that of our previously published studies or pilot studies. The assumed technical failure rate is based on previous experience. The method for calculating the number of required mice is according to Glantz and Slinker: *Primer of applied regression and analysis of variance*, McGraw-Hill, Inc. 2002.

**Replicates**

- You should report how often each experiment was performed
- You should include a definition of biological versus technical replication
- The data obtained should be provided and sufficient information should be provided to indicate the number of independent biological and/or technical replicates
- If you encountered any outliers, you should describe how these were handled
- Criteria for exclusion/inclusion of data should be clearly stated
- High-throughput sequence data should be uploaded before submission, with a private link for reviewers provided (these are available from both GEO and ArrayExpress)

Please outline where this information can be found within the submission (e.g., sections or figure legends), or explain why this information doesn’t apply to your submission:

The RNAseq data that were used to determine the PSI values are provided in an Excel spreadsheet and how often experiments had been repeated and biological and technical replicate information is in the captions. All data have been included.

**Statistical reporting**

- Statistical analysis methods should be described and justified
- Raw data should be presented in figures whenever informative to do so (typically when N per group is less than 10)
- For each experiment, you should identify the statistical tests used, exact values of N, definitions of center, methods of multiple test correction, and dispersion and precision measures (e.g., mean, median, SD, SEM, confidence intervals; and, for the major substantive results, a measure of effect size (e.g., Pearson's r, Cohen's d)
- Report exact p-values wherever possible alongside the summary statistics and 95% confidence intervals. These should be reported for all key questions and not only when the p-value is less than 0.05.

Please outline where this information can be found within the submission (e.g., sections or figure legends), or explain why this information doesn’t apply to your submission:

Figure 1. D) Two-way ANOVA with a Sidak’s multiple comparison analysis. Mean ± SEM, n-value 19, 5, 7, 8 for mice 60 d, 80d, 182d, and 274d, respectively.

Figure 2. B) Mean values of 8 WT and 8 Ttn^Δ112-158^ muscles from 60 d mice with randomly two muscles pooled, providing 4 samples per genotype. Mean PSI values are shown. P-values determined by t-tests.

Figure 3. Four muscles per genotype (from 4 mice 60 day old); 3 muscle strips from each muscle with ~ 50 data points per muscle. Linear regression analysis in B (R^2^ 0.7-0.9; slopes are significantly different (p<0.001)) and exponential growth curve in C (R^2^ 0.6-0.9). Insets: mean ± SEM, n= 11 and 16 in EDL in WT and Ttn^Δ112-158^ mice; ****: p<0.0001).

Figure 4. Mean ± SEM, n= 12 and 19 in Diaphragm, 15 and 11 in EDL and 5 and 5 in Soleus in 60 d old WT and Ttn^Δ112-158^ mice, respectively; inset t-test; ****: p<0.0001.

Figure 5. Functionally dissecting passive muscle stiffness.

A and B) Data from 9 mice (60 day) per genotype, Mean ± SEM; insets were tested with paired t-tests.

C) N-values for single fibers: 19 (WT) and 23 (Ttn^Δ112-158^ ) obtained from 5 mice (60 day old). Inset : two-way ANOVA with a Sidak’s multiple comparison analysis.

D) Two-way ANOVA with a Sidak’s multiple comparison analysis.

Figure 6. A) Mean ± SEM, n= 9 WT and 9 in Ttn^Δ112-158^ muscles.

B) Two-way ANOVA with a Sidak’s multiple comparison analysis.

C) Two-way ANOVA) with a Sidak’s multiple comparison. Mean ± SEM, n-values for B and C: Diaphragm: 13 and 10, EDL 6 and 5, EDL 5^th^ toe muscle 9 and 9, and Soleus 5 and 5 for WT and Ttn^Δ112-158^ , respectively.

Figure 7. A) Results from 5 mice, 60 d old. B) Mean ± SEM, n= 8 WT and 9 Ttn^Δ112-158^ mice. T-tests were used to test for significance.

C) Mean ± SEM, n= 8 WT and 9 Ttn^Δ112-158^ mice. T-tests were used to test for significance.

Figure 8. Two-way ANOVA with a Sidak’s multiple comparison analysis. Mean ± SEM, n= 5 WT and 5 Ttn^Δ112-158^ muscle with 5 fibers per muscle. A t-test was used to test for significance.

Figure 9. A-C) Two-way ANOVA (group sizes, 5-13). Mean ± SEM.

D) Two-way ANOVA. Mean ± SEM are shown.

Figure 10. A) Mean ± SEM are shown; results from 5 mice per genotype (60 d old) with 3 muscle strips per muscle and ~10 measurements per muscle strip.

(For large datasets, or papers with a very large number of statistical tests, you may upload a single table file with tests, Ns, etc., with reference to sections in the manuscript.)

**Group allocation**

- Indicate how samples were allocated into experimental groups (in the case of clinical studies, please specify allocation to treatment method); if randomization was used, please also state if restricted randomization was applied
- Indicate if masking was used during group allocation, data collection and/or data analysis

Please outline where this information can be found within the submission (e.g., sections or figure legends), or explain why this information doesn’t apply to your submission:

Group allocation was based on genotyping the mice. PCR-based genotyping was performed twice and was confirmed at the protein level using gels that allow us determine the genotype from the mobility of T1 on gels.

**Additional data files (“source data”)**

- We encourage you to upload relevant additional data files, such as numerical data that are represented as a graph in a figure, or as a summary table
- Where provided, these should be in the most useful format, and they can be uploaded as “Source data” files linked to a main figure or table
- Include model definition files including the full list of parameters used
- Include code used for data analysis (e.g., R, MatLab)
- Avoid stating that data files are “available upon request”

Please indicate the figures or tables for which source data files have been provided:

Source files have been provided for Fig. 2 and Figures S2A-C.
